# The Debilitating Physical and Emotional Effects of Limb Deformity: A Tertiary Center Observational Study Utilizing the Limb Deformity-Modified Scoliosis Research Society Score (LD-SRS)

**DOI:** 10.3390/jcm14030703

**Published:** 2025-01-22

**Authors:** Abdullah Addar, Fayez A. Alhabib, Nasser M. AbuDujain, Hani A. Alghamdi, Mohammed H. Alshalan, Hamza M. Alrabai, Fahad Alhuzaimi

**Affiliations:** 1 Department of Orthopedic Surgery, College of Medicine, King Saud University, Riyadh P.O. Box 11495, Saudi Arabia; 2 College of Medicine, Alfaisal University, Riyadh P.O. Box 11533, Saudi Arabia; 3 Department of Family and Community Medicine, College of Medicine, King Saud University, Riyadh P.O. Box 11495, Saudi Arabia; 4 Department of Family Medicine & Polyclinics, King Faisal Specialist Hospital and Research Centre, Riyadh P.O. Box 11211, Saudi Arabia

**Keywords:** limb deformity, quality of life, physical, emotional, LD-SRS, Saudi Arabia

## Abstract

**Background**: Limb deformity describes a condition where a limb has an abnormal shape or length, resulting from an acquired or a congenital case. This condition can impair the normal function of patients’ lives, leading to adverse psychological impacts. The limb deformity-modified Scoliosis Research Society (LD-SRS) score is a frequently used tool to assess quality of life. This observational study aimed to investigate the quality of life of patients with debilitating limb deformities in Saudi Arabia using the LD-SRS score. **Methods**: This is an observational analytical cross-sectional study conducted at the orthopedic clinic at King Saud University Medical City between March and May 2024. Patients diagnosed with any type of limb deformity (of different ages) were included in this study. Patients completed the LD-SRS score in the clinic and were divided into two groups based on whether they had undergone surgery for limb deformity. **Results**: This study included 152 participants, with the majority of participants in the 13–18-year age group and predominantly male (59.87%). Most participants were assessed before treatment, with significant differences observed in total scores based on the examination time (*p* < 0.001). Post-surgery scores showed marked improvement, with the overall total mean score for the surgery group being significantly higher than that of the non-surgery group (*p* < 0.001). Domain-specific scores revealed higher satisfaction in self-image/appearance for the surgery group, while the non-surgery group had lower scores in function/activity and mental health domains. No significant differences were found across age, gender, side of the body affected, or affected site. **Conclusions**: Preoperative patients had lower QoL scores as a whole group compared to those who had undergone surgery. The longer the duration postoperatively, the better the QoL score; those measured at three months had better QoL than preoperative patients, and those assessed at one and two years scored even higher. Self-image/appearance was a key factor, with the surgery group scoring higher in this domain than the non-surgery group. Future research should explore broader patient perspectives, including those related to mental and social well-being.

## 1. Introduction

Limb deformity describes a condition where a limb has an abnormal shape or length, resulting from an acquired or a congenital case [1]. Acquired cases arise from trauma, infections, and tumors, causing bone defects, malunion, or nonunion [1,2]. Congenital cases result from defects in fetal development, including angular deformities, rotational malalignment, and limb reduction defects [1,2]. The deformity’s physical aspect can impair patients’ normal function, leading to adverse psychological impact. We call this a “debilitating” condition, where the deformity causes a serious impairment of ability or function. Patients report depression, sleep problems, and sometimes dependence on others for daily activities [3]. The prevalence of such deformities is considerable, especially in younger ages. One cross-sectional descriptive study conducted in Egypt in 2021 aimed to determine the prevalence of limb deformities in primary school children in their governorate. The study included a total of 4689 primary school students, and the prevalence of lower limb deformity was estimated to be 16.61% among them [4].

The burden faced by patients with limb deformities ranges from impaired functionality and pain to psychological impact, all of which constitute quality of life (QoL). A study by Heath et al. [5] compared the QoL of patients with limb deformities with control subjects. They concluded that the patients had a worse QoL than the control subjects, regardless of the degree of deformity. The QoL of these patients is a significant factor in management, as poor QoL can serve as an indication for surgical intervention [5,6].

Detecting and addressing deformities early may have a profound impact on patients’ QoL and management [4]. Several patient-reported outcome measures (PROMs) are used to assess limb deformity, each focusing on different aspects. One used specifically in children is the LIMB-Q Kids [7].

Another PROM used specifically for assessing QoL in limb deformity is the limb deformity-modified Scoliosis Research Society (LD-SRS) score, a modified version of the Scoliosis Research Society (SRS) outcome measure [5]. It is designed for patients with a primary diagnosis of limb deformity and groups them into preoperative and postoperative populations while categorizing them based on the affected site, including the upper limb, thigh, leg, and foot/ankle. However, the tool does not account for individual variables such as specific diagnosis and treatment. The LD-SRS measures and scores five different domains: function/activity, pain, self-image/appearance, mental health, and satisfaction with management [8].

Several studies used LD-SRS scores to quantify patients’ deformity-related QoL [5,9,10,11], including pediatric patients [12]. To our knowledge, no investigations aiming to assess the QoL of patients with limb deformity have been published in the Middle East. Thus, this observational study aimed to investigate the quality of life of patients with debilitating limb deformities in the Saudi Arabian population using the LD-SRS score.

## 2. Methodology

### 2.1. Study Design, Participants, and Setting

This is an observational study conducted at King Saud University Medical City, Riyadh, Saudi Arabia, between March and May 2024. It is a cross-sectional study measuring patients at one point and dividing them into surgery and non-surgery groups. Data collection was conducted at the orthopedic clinic using a questionnaire, with a final sample size of 152 participants. We included adult and pediatric populations exclusively with limb deformity as the primary diagnosis, without collecting specific diagnoses. Patients followed up by the orthopedic clinic without deformity were not included in this study.

### 2.2. Instruments

Participants were given an electronic survey that included two parts. The first asked about demographic characteristics, and the second included the LD-SRS tool. The demographics section included questions about age, sex, time of examination (either before or after treatment and how long after), and affected limb and side.

#### LD-SRS

The LD-SRS tool is a patient-reported outcome measure currently being used to assess the quality of life of patients with a limb deformity. It was initially developed to evaluate the quality of life of scoliosis patients but has been modified for use in limb deformity [8]. The minimum and maximum scores are 1.0 and 5.0, respectively; the higher the score, the better the quality of life. The LD-SRS instrument has undergone various procedures to establish its reliability and validity. Reliability is evaluated based on internal consistency (0.977) and test–retest reliability (0.906), which confirm stable measurement across different domains and over time. The LD-SRS tool consists of 30 questions in 5 domains. Pain measures the intensity and frequency of pain related to the deformity and its effect on daily activities. Function/activity measures the limitations and struggles in everyday functions. Self-image/appearance is used to determine patients’ perception of their body image compared to others. Mental health questions are crucial to determine the quality of life, where adverse psychological impact could lead to symptoms of anxiety and depression. Satisfaction with management determines how patients perceive the effectiveness of the intervention they have undergone, if any. The LD-SRS was translated and validated for Arabic speakers, and it showed excellent psychometric properties in terms of internal consistency (0.95) and test–retest reliability (0.97) (in press).

### 2.3. Procedure and Data Collection

Participants were mainly recruited at the orthopedic limb deformity clinic for adult and pediatric populations using an electronic survey. Patients with limb deformities or their guardians were asked if they wanted to participate in this study. Details of this study were explained, including the aim, purpose, and right to refuse participation for any reason, after which the consent form was signed. Those accepted to be enrolled in the study were handed the electronic questionnaire to be filled out in the clinic using an electronic tablet.

All participants completed the first 20 questions, and an additional 10 questions were asked of those who had undergone limb surgery. For younger participants, the questionnaire was given to their guardians.

### 2.4. Ethical Considerations

This study was approved by the Institutional Review Board office of the College of Medicine at King Saud University, Riyadh, Saudi Arabia (approval no. E-23-8309, Ref. No. 23/0849/IRB). Consent was obtained electronically where each patient or the patient’s guardian was informed of the goal and purpose of this study, the principal investigator’s contact information, the right to withdraw from this study with no obligations, and the fact that participation would be anonymous with no identifying data being used. No incentives or rewards were given to the participants.

### 2.5. Statistical Data Analysis

The data were analyzed using R (version 4.2.2). Descriptive statistics were used to summarize sociodemographic characteristics, with frequencies and percentages presented for categorical variables. For the questionnaire data, domain scores and total scores were calculated to assess the overall and specific dimensions of patient outcomes. To compare the mean values of the domain and total scores across different patient characteristics, independent-sample *t*-tests were used for comparisons between two groups (e.g., gender, side of the body affected), and one-way analysis of variance (ANOVA) was employed for comparisons across multiple groups (e.g., age groups, time of examination). Post hoc tests were conducted where applicable to explore further significant differences identified with ANOVA. Additionally, *t*-tests were used to evaluate differences in mean scores for the first 20 questions between participants who had not undergone surgery and those who had. The last 10 questions were excluded from this comparison as they were designed to evaluate outcomes after completing treatment, which did not apply to the non-surgery group. Statistical significance was set at *p* < 0.05 for all analyses.

## 3. Results

- Sociodemographic characteristics

This study included 152 participants, with 25.66% (*n* = 39) in the surgery group and 74.34% (*n* = 113) in the non-surgery group, as shown in Table 1. The age of the participants varied across groups, with the majority falling in the 13–18-year age range (36.18%). The next largest group was aged 19–30 years, representing 27.63% of the total sample. The non-surgery group had more younger participants aged 0–12 years (23.89%) than the surgery group (15.38%), whereas the surgery group had a slightly higher percentage of older participants aged 31–50 years (15.38% vs. 11.50%). The sample was predominantly male (59.87%), with a similar distribution in both the surgery (61.54%) and non-surgery (59.29%) groups. Regarding the timing of the examination, the majority of participants (74.34%) were examined before any treatment. In the surgery group, 35.90% of the participants were examined two years after surgery, followed by 33.33% at three months after surgery. For the side of the body affected, the left side was more commonly affected in both groups, comprising 69.23% in the surgery group and 53.98% in the non-surgery group. As for the affected site, the most common area was the leg, comprising 37.50% of the total sample. This trend was consistent across both groups, although the surgery group had a higher prevalence of thigh deformities (41.03%) compared to the non-surgery group (32.74%). The foot/ankle was the next most affected site, followed by the upper limb, which was the least affected site overall.

- Score analysis based on patients’ characteristics

Table 2 provides the mean (SD) of total scores across various patient characteristics, along with the *p*-values from the ANOVA and *t*-tests used to assess statistical significance. The analysis revealed no statistically significant differences in total scores across age groups (*p* = 0.69), gender (*p* = 0.24), side of the body affected (*p* = 0.38), or affected site (*p* = 0.48). Specifically, the mean scores were relatively consistent across these categories, with minor variations that did not reach statistical significance. In contrast, a statistically significant difference was observed in total scores based on the time of examination (*p* < 0.001). Scores showed a marked and progressive increase from before treatment (mean = 68.2) to 3 months after treatment (mean = 107), with further improvements observed at 1 year (mean = 119) and 2 years after treatment (mean = 121).

- Comparison of domain-specific scores based on surgical status

Table 3 shows the mean and standard deviation for domain-specific scores across the total sample, as well as for patients who had undergone surgery compared to those who had not. The overall mean score for the total sample was 80 (SD = 27), with domain-specific mean values ranging from 2.0 (SD = 3.6) for satisfaction with management to 18.2 (SD = 6.2) for self-image/appearance. In the surgery group, the mean scores were higher across all domains, with the highest mean score observed for self-image/appearance (mean = 35.7, SD = 8.4) and the lowest for mental health (mean = 18.8, SD = 4.7). The total mean score for this group was 114.1 (SD = 22.9). For the non-surgery group, pain had the highest mean score (mean = 17.8, SD = 4.9). Notably, aside from satisfaction with management, which was expectedly low due to the lack of surgical intervention, the lowest mean score was observed for function/activity (mean = 16.3, SD = 4.5). The total mean score for the non-surgery group was 68.2 (SD = 16.1).

- Comparison of mean total scores based on surgical status and patient group

Table 4 presents a comparison between the total mean scores prior to surgery and overall scores among patients who had undergone surgery versus those who had not. For the Q1-20 scores, the non-surgery group (N = 113) had a mean (SD) of 68.2 (16.1) compared to 72.2 (16.3) in the post-surgery group (N = 39), with no statistically significant difference between the two groups (*p* = 0.19). However, the overall total scores showed a marked difference, with the non-surgery group scoring 68.2 (16.1) and the post-surgery group scoring 117 (22.9). This difference was statistically significant (*p* < 0.001).

## 4. Discussion

This study aimed to investigate the quality of life of patients with limb deformities using the patient-reported outcome measure LD-SRS. The most significant finding observed in our study was the difference in total scores based on time of examination (in relation to treatment). The LD-SRS scores in the non-surgery group showed a mean of 68.2, which progressively increased in the groups that had undergone surgery, meaning that those who had undergone surgery had better LD-SRS scores, as shown in Table 2. The first surgery group included patients who were examined three months after treatment (mean = 107), with further improvements in the mean score observed in the one-year group (mean = 119) and two-year group (mean = 121). Moreover, as shown in Table 4, the overall mean score of patients who had undergone surgical intervention was significantly higher than the non-surgery group’s mean. This indicates an enhanced quality of life for patients after surgical correction of limb deformity. These results highlight the importance of surgery not just for correcting the physical abnormality but also for ensuring long-term functional, emotional, and social well-being. Poor quality of life can be an indication of a need for medical and surgical interventions [5]. Our findings attest to the significance of such surgeries and their long-term effects.

For the non-surgery group, the lowest mean score observed was for satisfaction with management, an expected result due to the lack of surgical intervention and the continued impact of the deformity on the patients. In contrast, the surgery group had a higher score, indicating a positive patient perspective regarding the surgical correction, as shown in Table 3. The second lowest score in the non-surgery group was for self-image/appearance (mean = 16.1, SD = 4.7). This domain had the highest score in the surgery group (mean = 35.7, SD = 8.4), making it the domain with the largest difference in mean values between the two groups. This highlights the extent of patient dissatisfaction with their appearance and how much it affects their overall quality of life. Prior studies reported patients with signs of depression, anxiety, and self-consciousness related to appearance alone [3], resulting in a desire for surgical correction. The scores of the surgery group were shown to be the opposite in this domain, with great satisfaction after correction.

Amputation is a unique physical operation that needs to be differentiated from limb deformity. A study by Puranik et al. described the QoL and long-term outcomes of patients with major amputations [13]. Although usually performed as a life-saving procedure, patients reported unfavorable consequences on mental, physical, and social well-being. These poor outcomes were explained by the sudden change in lifestyle due to new physical limitations, which led to further mental strains. Despite this, most patients reported significant improvement in QoL from two to six months postoperatively. Improving the management of the resultant limitations resulted in improved physical and mental well-being, as well as heightened self-esteem. Such results are also reflected in our study, where we found progressive improvement in QoL from patients examined three months after treatment to patients examined two years after treatment.

Based on our findings and as highlighted by Benes et al. [14] in their 2024 publication, socioeconomic deprivation was identified as a significant factor influencing limb deformity progression and poor prognosis. The data further supported this, showing that Black patients with Blount disease experienced higher socioeconomic deprivation and worse mechanical axis deviation. Additionally, multivariate analysis revealed that increased body mass index and state-level socioeconomic deprivation were independent predictors of more severe varus deformity, underscoring the critical role of social determinants in disease outcomes. Furthermore, considerable psychological distress has been noted to follow severe limb deformities and injury [15], and a large body of literature confirms the negative mental health impact mainly experienced by amputees [16,17,18].

Although King Saud University Medical City receives patients from different parts of Saudi Arabia, it is difficult to generalize our results to the whole patient population as the data were only collected from one center. Our study also lacked detailed information on the patient population, as we only included patients with limb deformity as their primary diagnosis, regardless of the type. More details about the type of deformity would have helped clarify the results as some conditions naturally lead to a much poorer QoL than others. We acknowledge the potential impact of heterogeneity on our findings. Our study analyzed patients’ LD-SRS scores for up to two years after treatment or surgical correction. This limitation could have been addressed to enhance the generalizability of the results. Future studies should aim to include multiple centers with a more extensive study population and analyze more variables, such as socioeconomic impact and the role of mental health in relation to limb deformity. An analysis based on the type of deformity will also advance the understanding of specific deformities and their effect on QoL, as well as the urgency of management. Furthermore, we recommend that future studies investigate patient responsiveness within more clearly defined subgroups. This could include stratified analyses based on factors such as deformity type or severity, as well as assessments of pretreatment self-image perceptions, gender-based differences, and low preoperative satisfaction in non-surgery patients.

## 5. Conclusions

Preoperative patients had lower QoL scores as a whole group compared to those who had undergone surgery. The longer the duration postoperatively, the better the QoL score; those assessed at three months had better QoL scores than preoperative patients, and those assessed at one and two years scored even higher. The overall mean of the surgery group was also significantly higher than that of the non-surgery group, indicating an improved quality of life. The lowest domain score found in the non-surgery group was for self-image/appearance. In contrast, this was the highest domain score in the surgery group, which greatly impacted the overall QoL. Future research is needed to better understand patient perspectives that extend beyond addressing physical aspects. By analyzing quality of life, research can promote innovations that not only improve functional outcomes but also enhance mental and social well-being.

## Figures and Tables

**Table 1:** Demographic characteristics.

Characteristics		N (%) Total 152	Surgery (39)	Non-Surgery (113)
Age	0–12	33 (21.71)	6 (15.38)	27 (23.89)
	13–18	55 (36.18)	16 (41.03)	39 (34.51)
	19–30	42 (27.63)	11 (28.21)	31 (27.43)
	31–50	19 (12.50)	6 (15.38)	13 (11.50)
	>50	3 (1.97)	0	3 (2.65)
Gender	Male	91 (59.87)	24 (61.54)	67 (59.29)
	Female	61 (40.13)	15 (38.46)	46 (40.71)
Time to examination	Before treatment	113 (74.34)	0	113 (100)
	3 months after treatment	13 (8.55)	13 (33.33)	0
	6 months after treatment	4 (2.63)	4 (10.26)	0
	1 year after treatment	8 (5.26)	8 (20.51)	0
	2 years after treatment	14 (9.21)	14 (35.90)	0
Side	Left	88 (57.89)	27 (69.23)	61 (53.98)
	Right	64 (42.11)	12 (30.77)	52 (46.02)
Affected site	Upper limb (hand, wrist, forearm, arm)	9 (5.91)	2 (5.12)	7 (6.19)
	Foot/ankle	33 (21.71)	8 (20.51)	25 (22.12)
	Leg	57 (37.50)	13 (33.33)	44 (38.94)
	Thigh	53 (34.87)	16 (41.03)	37 (32.74)

**Table 2:** Mean (SD) of total scores across various patient characteristics.

Characteristics		Overall Sample		Adult Patients			Pediatric Patients		
		Mean (SD)	*p*-Value		Mean (SD)	*p*-Value		Mean (SD)	*p*-Value
Age	0–12	80 (26.7)	0.69 ANOVA	17–25	77 (26)	0.65	2–5	70.8 (17.2)	0.56
	13–18	78.9 (24.6)		26–35	82.8 (33.3)		6–9	86.2 (30.5)	
	19–30	78.1 (28.5)		36–65	85.5 (29.6)		10–13	77.7 (23.9)	
	31–50	88.4 (32.4)					14–17	78.4 (24.6)	
	>50	73.7 (22)							
Gender	Male	82.1 (27)	0.24 *t*-test	88.8 (29)		**0.01**	77.6 (24.9)		0.39
	Female	76.8 (26.9)		70.1 (26.6)			82.5 (26.2)		
Time to examination	Before treatment	68.2 (16.1)	**<0.001** ANOVA	67.1 (16.5)		**<0.001**	69.1 (15.9)		**<0.001**
	3 months after treatment	107 (16.7)		109 (20.1)			107 (16.8)		
	6 months after treatment	102 (35.9)		71 (n.a)			113 (35.8)		
	1 year after treatment	119 (12)		117 (10.8)			126 (17.7)		
	2 years after treatment	121 (27.4)		133 (20.5)			109 (29.8)		
Side	Left	81.6 (28.4)	0.38 *t*-test	83.6 (34.2)		0.39	80.3 (24.1)		0.71
	Right	77.8 (25.1)		77.4 (22.4)			78.1 (27.5)		
Affected site	Upper limb (hand, wrist, forearm, arm)	82.1 (30.8)	0.48 ANOVA	118 (39.6)		0.14			0.48
	Foot/ankle	82.6 (25)		81.3 (28.8)			83.3 (23.6)		
	Leg	75.5 (27.6)		72.8 (25.6)			78.6 (29.8)		
	Thigh	82.8 (27.1)		87.8 (31.7)			79.5 (23.6)		

**Table 3:** Mean and standard deviation for domain-specific scores for the total sample, and patients who had undergone surgery versus those who had not.

Domains	Mean (SD) All	Mean (SD) Surgery	Mean (SD) Non-Surgery
Function/activity	16.5 (4.5)	24.7 (5.9)	16.3 (4.5)
Pain	18 (4.8)	22.7 (5.2)	17.8 (4.9)
Self-image/appearance	18.2 (6.2)	35.7 (8.4)	16.1 (4.7)
Mental health	18.2 (4.9)	18.8 (4.7)	17.9 (4.9)
Satisfaction with management	2 (3.6)	12.1 (2.9)	0
Total score	80 (27)	114.1 (22.9)	68.2 (16.1)

**Table 4:** Comparison between total mean scores prior to surgery and after treatment among patients who had undergone surgery vs. non-surgery patients.

Surgical Status	Overall Sample					Adult Patients Only		Pediatric Patients Only	
Timing	N	Q1-20 Mean (SD)	*p*-Value	Overall Total Mean (SD)	*p*-Value	Total Mean (SD)	*p*-Value	Total Mean (SD)	*p*-Value
Non-surgery	113	68.2 (16.1)	0.19	68.2 (16.1)	<0.001	67.1 (16.5)	<0.001	69.1 (15.9)	<0.001
Post-surgery	39	72.2 (16.3)		117 (22.9)		119 (22.1)		110 (23.2)	

## Data Availability

Data will be shared with request to the corresponding author.

## References

[1] Chhina H., Klassen A., Kopec J., Park S., Fortes C., Cooper A. (2017). Quality of Life of Children with Lower Limb Deformities: A Systematic Review of Patient-Reported Outcomes and Development of a Preliminary Conceptual Framework. J. Limb Lengthening Reconstr..

[2] Chhina H., Klassen A.F., Kopec J.A., Oliffe J., Iobst C., Dahan-Oliel N., Aggarwal A., Nunn T., Cooper A.P. (2021). What matters to children with lower limb deformities: An international qualitative study guiding the development of a new patient-reported outcome measure. J Patient Rep. Outcomes.

[3] Ramaker R.R., Lagro S.W., van Roermund P.M., Sinnema G. (2000). The psychological and social functioning of 14 children and 12 adolescents after Ilizarov leg lengthening. Acta Orthop. Scand..

[4] Ganeb S.S., Egaila S.E.S., Younis A.A., El-Aziz A.M.A., Hashaad N.I. (2021). Prevalence of lower limb deformities among primary school students. Egypt. Rheumatol. Rehabil..

[5] Heath M.R., Shin T.J., Mehta R., Principe P.S., Mackie A.T., Fragomen A., Rozbruch S.R., Fabricant P.D. (2021). Patients with Lower Limb Deformity Report Worse Quality of Life than Control Subjects Regardless of Degree of Deformity. J. Am. Acad. Orthop. Surg. Glob. Res. Rev..

[6] Montpetit K., Hamdy R.C., Dahan-Oliel N., Zhang X., Narayanan U.G. (2009). Measurement of Health-related Quality of Life in Children Undergoing External Fixator Treatment for Lower Limb Deformities. J. Pediatr. Orthop..

[7] Chhina H., Klassen A., Bade D., Kopec J., Cooper A. (2022). Establishing content validity of LIMB-Q Kids: A new patient-reported outcome measure for lower limb deformities. Qual. Life Res..

[8] Fabricant P.D., Borst E.W., Green S.A., Marx R.G., Fragomen A.T., Rozbruch S.R. (2016). Validation of a modified Scoliosis Research Society instrument for patients with limb deformity: The limb deformity-Scoliosis Research Society (LD-SRS) score. J. Limb Lengthening Reconstr..

[9] Tsutsui S., Pawelek J., Bastrom T., Lenke L., Lowe T., Betz R., Clements D., Newton P.O. (2009). Dissecting the effects of spinal fusion and deformity magnitude on quality of life in patients with adolescent idiopathic scoliosis. Spine.

[10] Brune C.S., Toporowski G., Rölfing J.D., Gosheger G., Fresen J., Frommer A., Laufer A., Roedl R., Vogt B. (2022). German Translation and Cross-Cultural Adaptation of the Limb Deformity-Scoliosis Research Society (LD-SRS) Questionnaire. Healthcare.

[11] Greenstein M.D., Ellsworth B.K., Sheridan G.A., Fragomen A.T., Rozbruch S.R. (2023). Signficant Femoral Version Abnormalities and Patient-Reported Quality of Life. J. Am. Acad. Orthop. Surg. Glob. Res. Rev..

[12] Sabharwal S., Coufal S., Less J., Sabharwal S. (2024). Concurrent Validity of PROMIS and LD-SRS Scores in Pediatric Patients with Lower Limb Differences. J. Pediatr. Orthop..

[13] Puranik A.K., Maity S., Meena S.P., Santra A., Lodha M., Badkur M. (2021). Quality of Life of Patients with Major Amputations in the Tertiary Care Center of Western Rajasthan: A Prospective Observational Study in 2019–2020. Cureus.

[14] Benes G., Ghanem D., Badin D., Greenberg M., Honcharuk E. (2024). The Effect of Socioeconomic Deprivation on Radiographic Deformities in Children with Blount Disease. J. Pediatr. Orthop..

[15] McCarthy M.L., MacKenzie E.J., Edwin D., Bosse M.J., Castillo R.C., Starr A., LEAP study group (2003). Psychological distress associated with severe lower-limb injury. J. Bone Jt. Surg. Am..

[16] Şimsek N., Öztürk G.K., Nahya Z.N. (2020). The Mental Health of Individuals with Post-Traumatic Lower Limb Amputation: A Qualitative Study. J. Patient Exp..

[17] Jo S.H., Kang S.H., Seo W.S., Koo B.H., Kim H.G., Yun S.H. (2021). Psychiatric understanding and treatment of patients with amputations. Yeungnam Univ. J. Med..

[18] Krijgh D.D., Teunis T., List E.B., Mureau M.A.M., Luijsterburg A.J.M., Maarse W., Schellekens P.P.A., Hietbrink F., de Jong T., Coert J.H. (2024). Mental health is strongly associated with capability after lower extremity injury treated with free flap limb salvage or amputation. Eur. J. Trauma Emerg. Surg..

